# Enhancing academic success in higher education: the predictive role of critical thinking through sequential mediation of academic control, self-efficacy, and intrinsic motivation

**DOI:** 10.3389/fpsyg.2026.1783512

**Published:** 2026-05-19

**Authors:** Ema Lukačková, Gabriela Šeboková

**Affiliations:** Department of Psychological Sciences, Faculty of Social Sciences and Health Care, Constantine the Philosopher University in Nitra, Nitra, Slovakia

**Keywords:** academic control, academic success, critical thinking, higher education, intrinsic motivation, self-efficacy, sequential mediation

## Abstract

**Introduction:**

Academic success (AS) represents a central outcome of contemporary education; however, this complex construct is often narrowly operationalized in the literature as a grade point average (GPA) alone. One factor increasingly regarded as essential for meeting current academic demands is critical thinking (CT), particularly in an era of easy access to information and misinformation. Nevertheless, the relationship between CT and AS remains unclear, and both constructs require more comprehensive examination, which is still limited in the existing literature. This study investigated the effects of CT skills and dispositions on AS, operationalized as GPA and perceived academic success, while examining academic locus of control, self-efficacy, and intrinsic motivation as sequential mediating mechanisms.

**Methods:**

The sample consisted of 263 university students aged 18–28 years (M = 21.74; SD = 1.56). Data were collected using a short version of the Watson–Glaser Critical Thinking Appraisal (WGCTA), the Critical Thinking Disposition Scale (CTDS), selected dimensions of the College Learning Effectiveness Inventory, the Perceived Academic Control Scale, the Intrinsic Goal Orientation (IGO) subscale of the Motivated Strategies for Learning Questionnaire (MSLQ), the General Academic Self-Efficacy Scale, and GPA. Four sequential mediation models were tested.

**Results:**

The results showed that both CT skills and dispositions significantly predicted perceived AS, including indirect effects through the mediators examined. GPA was directly predicted only by CT skills, whereas the indirect effects of both skills and disposition on GPA emerged solely through academic control.

**Discussion:**

These findings highlight the importance of CT and social cognitive factors in student retention and success in higher education. Simultaneously, they underscore the complex and psychosocial nature of AS, extending beyond traditional performance-based indicators.

## 1 Introduction

We live in an era of information revolution, which brings rapid access to information but also the risk of misinformation and manipulation. The inability to distinguish between true and false information affects more than half of the population (Newman et al., 2024) and undermines both rational judgment and trust in scientific institutions (Pillai and Fazio, 2021; Merva et al., 2025). In this context, the development of critical thinking (CT) has become an essential prerequisite for navigating the contemporary world. CT is a multidimensional construct encompassing both the cognitive skills necessary for analysis and evaluation and the dispositional characteristics that determine an individual's willingness to use these skills (Facione, 2000). Although this distinction between skills and dispositions is widely accepted, many studies have focused on only one component, overlooking the other. However, research on academic environments has predominantly focused on skills (Fong et al., 2017; Orhan, 2022). Such an approach may fail to capture the complexity of CT, as its effective functioning requires not only the presence of cognitive skills but also the disposition to apply them actively. Therefore, in this study, CT is conceptualized as an integrated construct that includes both skills and dispositions.

The development of CT is the primary goal of modern education and an important competence that supports academic success (AS; EHEA, 2024; Hamilton and Parsi, 2022). Academic success is often reduced to grade point average (GPA); however, this understanding does not provide a comprehensive view of the factors that determine true student achievement (Alyahyan and Düştegör, 2020; Kahu et al., 2017; York et al., 2015). Despite the growing theoretical recognition of academic success as a multidimensional construct, empirical research continues to rely predominantly on GPA as a proxy measure, creating a mismatch between conceptualization and operationalization (Rice et al., 2025). Recent evidence further highlights that student success encompasses multiple domains, including academic progress, satisfaction, skill development, and career outcomes, yet lacks a consistently applied empirical framework (Vugteveen et al., 2025). This limitation is particularly evident in the underrepresentation of the psychosocial aspects of academic functioning, despite their importance in student engagement, retention, and overall academic experience. Given the multidimensional nature of both variables, their relationship is inherently complex and shaped by multiple factors. Although the literature generally suggests a positive association between CT and AS, this relationship is inconsistent (Ross et al., 2013; Fong et al., 2017; Orhan, 2022), highlighting the need to examine the underlying mechanisms.

Socio-cognitive factors, including locus of control, self-efficacy, and motivation, have consistently been identified as important determinants of both critical thinking and academic success (Haidari et al., 2023; Heidari Gorji et al., 2018; Stupnisky et al., 2008). Despite their established relevance, existing research has predominantly examined these constructs in isolation, with limited attention paid to their combined or interactive effects on academic success. Meta-analytic evidence further suggests a lack of integrative approaches that would capture their joint contribution (Haidari et al., 2023). Conceptually, these variables can be subsumed under a broader framework of perceived control, indicating that they are theoretically interconnected. However, the potential sequential or structural relationships between them in the context of academic success remain underexplored.

Despite growing interest, CT and AS have still not been studied comprehensively. The relationship between these factors remains ambiguous (Ross et al., 2013; Fong et al., 2017; Orhan, 2022), and only a limited number of studies have focused on the psychosocial aspects of AS (Alyahyan and Düştegör, 2020; Kahu et al., 2017; York et al., 2015). CT is more often studied through skills than through dispositions (Fong et al., 2017; Orhan, 2022), and a comprehensive examination of all three components of perceived control is rare in the literature (Heidari et al., 2023). Therefore, this study aims to examine the influence of CT (skills and dispositions) on AS (GPA and perceived) and to test whether perceived control, through its three components, sequentially mediates this relationship—i.e., whether CT affects locus of control, which influences self-efficacy, which subsequently affects intrinsic motivation, ultimately supporting both perceived and GPA-based academic success.

### 1.1 Literature review

#### 1.1.1 Critical thinking and academic success: its relationship

Critical thinking has a rich history and multiple conceptualizations. The most widely recognized definition is that of Facione (1990), which has been further elaborated by other authors (Halonen, 1995; Halpern, 1998), who conceptualized it as purposeful, self-regulatory judgment involving interpretation, analysis, evaluation, and inference, as well as the explanation of the evidential, conceptual, methodological, criterial, or contextual considerations upon which such judgment is based. A distinction between skills and dispositions was introduced to explain the gap between potential and actual performance. An individual may possess the necessary analytical and reasoning skills; however, without the willingness to apply them, critical thinking may not manifest in practice. However, some authors have not explicitly distinguished between skills and dispositions. Kuhn (1999) conceptualizes critical thinking as a developmental process closely linked to metacognition and argumentation, whereas Chaffee (2011) defines it as an active process of evaluating information and judgments, implicitly encompassing both skills and attitudes. Empirical evidence indicating low-to-moderate correlations between critical thinking skills (CTS) and dispositions (Li et al., 2024; Yang and Chou, 2008) supports the view that CTS is a relatively independent construct. This distinction has important implications, as the development of one component does not necessarily translate into the advancement of the other. Consequently, a discrepancy may emerge between an individual's potential and their actual enactment, whereby students may possess well-developed cognitive skills yet fail to apply them in practice. In higher education, this misalignment may manifest as a divergence between academic performance and deeper engagement in learning. In the context of the labor market, this may be reflected in the gap between formal qualifications and the capacity to adaptively solve complex, real-world problems. Taken together, these considerations underscore the importance of examining critical thinking as a multifaceted construct that encompasses both skills and dispositions.

CT skills are deliberate, self-regulatory cognitive processes used to interpret, analyze, evaluate, and infer reasoning (Facione, 2000). Several authors (Halpern, 1998; Ku and Ho, 2010; Butler et al., 2012), including the RED (Recognize assumptions, Evaluate arguments, Draw conclusions) model (Watson, 2010), build on or simplify broader conceptions into practically measurable frameworks. They emphasize three key dimensions—recognizing assumptions, evaluating arguments, and drawing conclusions—that allow the understanding of information, assessment of its quality, and reasoning. Mueller et al. (2020) highlight that interpretation and evaluating arguments are particularly important in the academic context. Dispositions reflect internal motivations and tendencies toward engaging in critical thinking. Without them, individuals are unlikely to utilize their skills (Facione, 2000); however, research has predominantly focused on these skills (Fong et al., 2017; Orhan, 2022). Although Facione (2000) originally distinguishes multiple dispositional dimensions, several authors (Ku and Ho, 2010; Ennis, 2011; Sosu, 2013; Stanovich and Toplak, 2023) agree that truth-seeking and open-mindedness are key components supporting the activation of cognitive skills. Sosu (2013) further describes reflective skepticism (equivalent to truth-seeking) as the tendency to seek reliable information, question one's beliefs, and require evidence before concluding, whereas critical openness denotes the willingness to consider alternative perspectives and accept contradictory evidence.

AS is commonly conceptualized as a multidimensional construct encompassing not only performance but also satisfaction, emotional experience, and broader aspects of student functioning (Kim et al., 2010). In line with the widely cited framework of York et al. (2015), AS includes academic achievement, attainment of learning objectives, skill acquisition, satisfaction, persistence, and post-college outcomes. Despite its comprehensive nature, this model has been criticized for its limited practical applicability, as empirical studies typically operationalize only selected components—most commonly academic performance (e.g., GPA) and satisfaction (Guszak and Bazdan, 2023; Budiwati et al., 2024). Importantly, performance-based AS, expressed in grades or GPA, remains a key indicator of educational quality, as it determines academic progression (Moss and Yeaton, 2015), which likely explains its continued dominance in empirical research. At the same time, this tendency has motivated efforts to refine the construct. For instance, Vugteveen et al. (2025) identified five core dimensions of student success—academic progress, performance, goal attainment (including skills), satisfaction, and career outcomes—largely corresponding to York et al.'s (2015) framework, while offering a more parsimonious structure. Recent empirical work also supports a broader conceptualization of AS, highlighting the relevance of psychosocial dimensions alongside performance indicators (Rice et al., 2025). Accordingly, the present study distinguishes between GPA academic success as an objective performance measure and perceived academic success as a subjective psychosocial aspect of student functioning. The importance of studying CT in the academic environment has also been confirmed by a growing number of studies investigating this relationship. Several meta-analyses have shown that the development of CT disposition and skills is associated with GPA-based AS (Ross et al., 2013; Fong et al., 2017; Orhan, 2022). However, this relationship is not entirely unequivocal. First, far fewer studies have examined the relationship between CT disposition (CTD) and AS than those focusing on skills (Fong et al., 2017; Orhan, 2022). Second, not all studies report significant associations for either dispositions (Azar, 2010; Mahmoud, 2012; Tafazzoli et al., 2015) or skills (Singleton-Williams, 2009; Mohammadi et al., 2016; Shirazi and Heidari, 2019). Third, as noted, the psychosocial component of AS is often overlooked and has thus been studied much less. Nevertheless, evidence exists for positive associations between CT disposition and satisfaction with research courses (Landa-Blanco and Cortés-Ramos, 2021), interest in learning (Tong et al., 2023), and academic grit (Yüce, 2023). Higher CT dispositions also predict higher levels of major satisfaction (Eom et al., 2019). Similarly, positive relationships have been found between CT skills and student satisfaction (Al-Zarfi et al., 2024), as well as between CT skills and satisfaction with studies and academic experience (Huamán-Tapia et al., 2023; Shcheglova et al., 2024). Fourth, it is plausible that the relationship between CT and AS is mediated by other variables, several of which have been identified, including negative emotions (Villavincencio, 2011), self-efficacy (Liu et al., 2023), meaning of life (Celik et al., 2015), and rational beliefs (Balkis, 2013). In line with these considerations, the following relational hypotheses (H1a–H1d) are proposed.

CT dispositions significantly positively predicted GPA AS (H1a) and perceived AS (H1b), and CT skills significantly positively predicted GPA AS (H1c) and perceived AS (H1d).

#### 1.1.2 Academic control, self-efficacy, and intrinsic motivation as mediators in the proposed model

The construct closely related to CT and AS was perceived control. According to Wallston et al. (1987), its focus can be on outcomes, behaviors, or processes, encompassing locus of control, self-efficacy, and motivation (Perry et al., 2001; Skinner, 2014; Schunk and DiBenedetto, 2020). In social cognitive learning theory, these three variables play a fundamental role. Bandura (1986) emphasizes that belief in the ability to influence outcomes (locus of control) strengthens belief in one's capability to successfully perform tasks (self-efficacy), which positively affects motivation to act. Motivation, in turn, arises from satisfaction with performance and reinforces locus of control, thus closing the cycle of perceived control. These three constructs can therefore be collectively referred to as social cognitive aspects (Bandura, 1986). Their predictive value increases when operationalized according to the context they are intended to explain (Bandura, 1986). In this study, locus of control is defined as academic control (Perry et al., 2001), understood as a key component of the attribution process, specifically as a set of beliefs regarding the causes of success and failure (Perry et al., 2001). Self-efficacy is defined as general academic self-efficacy (Nielsen et al., 2018). In the case of intrinsic motivation in an academic context, we rely on the theoretical framework of Pintrich (1991), who refer to this construct in their questionnaire as Intrinsic Goal Orientation. The author considers it a key component of motivational orientations and equivalent to the traditional understanding of intrinsic motivation, where an activity is performed for one's own satisfaction.

These three constructs share several common characteristics, and meta-analytic findings suggest that they represent key variables for understanding academic performance and student behavior (Colquitt et al., 2000). As academic control, self-efficacy, and motivation are conceptually related and mutually interacting, their relationships appear reciprocal (Elliott and Del Puerto, 2014). However, both theoretical and empirical evidence support a sequential pathway among them, specifically in the order: academic control → self-efficacy → motivation. Academic control can be considered the initial variable, as it has been identified as a strong predictor of academic achievement, indicating its primary role in the psychological processes preceding motivation and performance (Oshakuade et al., 2023). A consistent link between academic control and self-efficacy has been established; repeated experiences of control over outcomes strengthen individuals' perceptions of their own competence (Anderson et al., 2005; Elliott and Del Puerto, 2014; Papoulidi and Maniadaki, 2025). Furthermore, both academic control and self-efficacy have consistently been shown to predict motivation (Anderson et al., 2005; Haidari et al., 2023). Heidari et al. (2023) suggest that locus of control influences academic achievement only indirectly through motivation, indicating that control beliefs alone may be insufficient for academic engagement. Instead, they shape students' expectations and motivation, which subsequently translate into performance, while their interplay with self-efficacy remains crucial, as low confidence leads students to avoid tasks despite attainable success.

Building on this socio-cognitive framework, although these three mediators are theoretically closely linked to CT, their relationships remain complex and not fully defined. In the existing literature, the relationship is more commonly examined in the direction where academic control, self-efficacy, and motivation predict CT dispositions (Stupnisky et al., 2008; Semerci, 2011; Tasgin and Dilek, 2023), as well as CT skills (Arif et al., 2025; Valenzuela et al., 2023; Agoes Salim et al., 2024). However, given that perceived control constructs and CT are multifaceted psychological phenomena, their relationship cannot be reduced to a unidirectional pathway. Evidence increasingly points to reciprocity, with CT also functioning as an antecedent predictor of academic control [Stupnisky et al., 2008 (dispositions); Flor et al., 2013 (skills)], self-efficacy [Çelikkol and Konik, 2023; Yurt and Hayli, 2025; Heidari et al., 2023 (dispositions); Heidari Gorji et al., 2018 (skills)], and intrinsic motivation [Kuloglu and Orhan, 2024 (dispositions); Points III, 2024 (skills)]. This perspective extends the prevailing body of research by capturing the dynamic and mutually reinforcing nature of these constructs and motivating a more integrative examination of their interactions.

**Figure 1:**
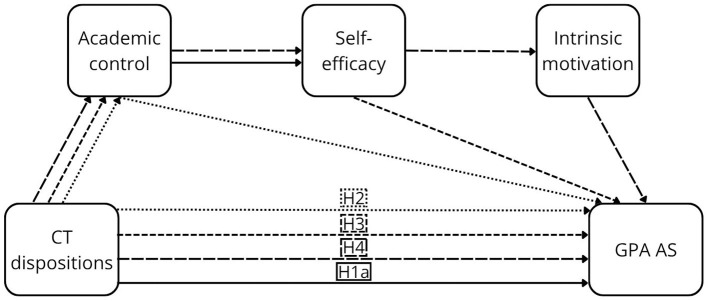
The proposed conceptual model of the relationship between CTD and GPA AS.

**Figure 2:**
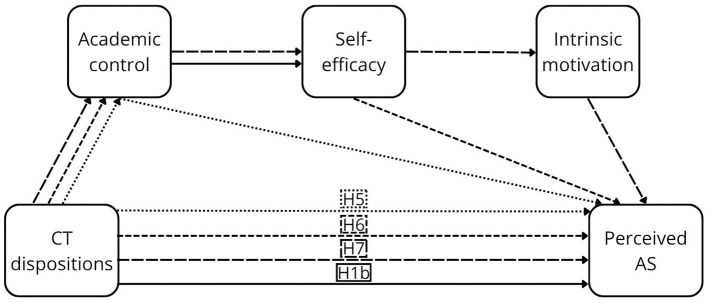
The proposed conceptual model of the relationship between CTD and perceived AS.

**Figure 3:**
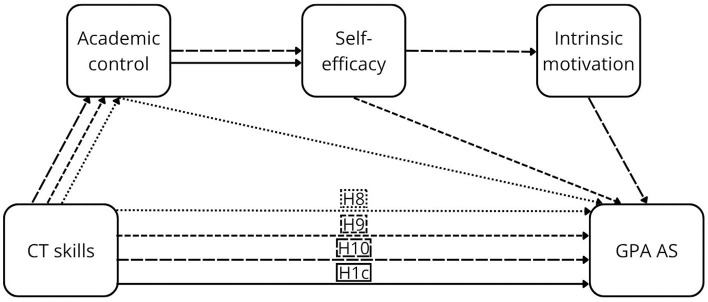
The proposed conceptual model of the relationship between CTS and GPA AS.

**Figure 4:**
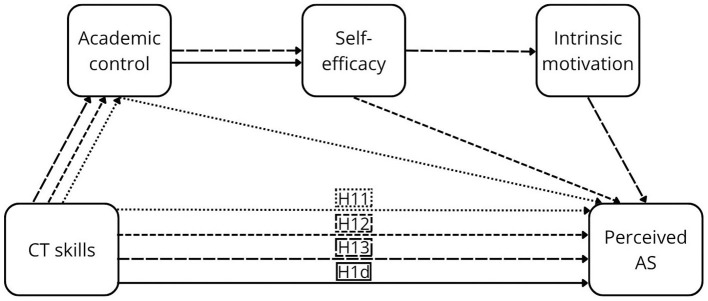
The proposed conceptual model of the relationship between CTS and perceived AS.

As noted above, the components of perceived control are central in academic contexts, with strong empirical support. Their positive effects on GPA AS have been consistently demonstrated in meta-analyses, particularly for academic control (Richardson et al., 2012), as well as self-efficacy and motivation (Haidari et al., 2023; Qi et al., 2024). This evidence was further strengthened by longitudinal meta-analytic findings that demonstrated stability and directional effects over time (Vu et al., 2024). Evidence regarding perceived AS is limited; however, insights from the broader domain of perceived outcomes indicate that student satisfaction—closely related to perceived AS—is consistently predicted by all components of perceived control (Alzukari, 2024; Chahal et al., 2025; Dumitrescu, 2016; Rustamov et al., 2024). In addition, individual components have been linked to other forms of perceived AS, including perceived stress (academic control: Dumitrescu, 2016; self-efficacy: Chahal et al., 2025; Dumitrescu, 2016), subjective academic success (self-efficacy: Alzukari, 2024; motivation: Alzukari, 2024), and students' academic experience (motivation: Kasemy et al., 2022). Detailed descriptions of the individual pathways are provided in Appendix A. Furthermore, mediation hypotheses (H2–H13) are proposed (see Figures 1–4).

Academic control mediates the relationship between CT dispositions and GPA AS (H2) and perceived AS (H5), as well as between CT skills and GPA AS (H8) and perceived AS (H11).

Academic control and self-efficacy sequentially mediate the relationship between CT dispositions and GPA AS (H3) and perceived AS (H6), as well as between CT skills and GPA AS (H9) and perceived AS (H12).

Academic control, self-efficacy, and motivation sequentially mediate the relationship between CT dispositions and GPA AS (H4) and perceived AS (H7), as well as between CT skills and GPA AS (H10) and perceived AS (H13).

## 2 Materials and methods

### 2.1 Participants

The minimum required sample size was estimated using a Monte Carlo simulation in R, based on a serial mediation model with assumed path coefficients of 0.30 from the independent variable to each mediator, 0.50 between mediators, and 0.30 from each mediator to the dependent variable. Using a target power of 0.80, the simulation indicated that a minimum of 168 participants were required (Thoemmes et al., 2010). The test battery was completed by 294 Slovak students. Based on a control question placed in the middle of the battery (“If you are still present, select option 7”), 31 responses were excluded. The final sample consisted of 263 university students in the humanities (213 female, 199 undergraduate) aged 18–28 years (M_age_ = 21.74, SD = 1.56). Ten students did not report their GPA in the questionnaire and were therefore excluded from the analyses involving this variable.

### 2.2 Measures

#### 2.2.1 Measure of critical thinking

To measure critical thinking (CT) skills, we used a shortened version of the Watson–Glaser Critical Thinking Appraisal (WGCTA II, Form C) validated in the Slovak population (Šeboková et al., 2026).

Selection combined empirical (e.g., factor loadings, model fit, reliability, and item–total correlations) and theoretical criteria, resulting in a nine-item unidimensional version with adequate model fit and acceptable reliability confirmed in an independent sample (Šeboková et al., 2026).

The selected items represented two dimensions of the original test: Interpretation (five items) and Evaluation of Arguments (four items). Interpretation reflects the ability to understand and accurately interpret the meaning of information and statements and consists of five items with dichotomous (yes/no) response options. The evaluation of arguments reflects the ability to assess the relevance and strength of arguments and consists of four items with dichotomous response options (strong vs. weak argument). The use of the shortened version was motivated by practical considerations, including time constraints and the need for efficient assessment in research and educational contexts.

CT dispositions were measured using the self-report questionnaire, Critical Thinking Disposition Scale (CTDS; Sosu, 2013), which measures two dimensions: reflective skepticism and critical openness. Critical openness (seven items) measures the tendency to be open to new ideas, to critically evaluate them, and to modify one's thinking based on compelling evidence. Reflective skepticism (four items) expresses the tendency to learn from past experiences and to be skeptical of evidence. The statements were rated on a five-point Likert scale.

#### 2.2.2 Measure of academic success

Perceived AS was measured using selected dimensions [which theoretically fit York et al.'s (2015) conceptual framework] from the self-report questionnaire, the College Learning Effectiveness Inventory (CLEI; Newton et al., 2008), on a five-point Likert scale. The Organization and Attention to Study Scale (eight items) reflects how students organize tasks and allocate time for academic activities. The Stress and Time Press Scale (six items) measures how students cope with time pressure, environmental issues, and academic demands that affect their studies. The Emotional Satisfaction Scale (seven items) expresses the level of interest and emotional response to academic life, including people and the educational environment at the university. The Class Communication Scale (six items) measures students' verbal and non-verbal efforts to engage in classroom activities.

GPA AS was measured based on the weighted grade point average (GPA) from the previous semester.

#### 2.2.3 Measure of academic control

Academic control was measured using the Perceived Academic Control Scale (PACS; Perry et al., 2001), which consists of eight items assessing the perceived ability to influence academic achievement outcomes rated on a five-point Likert scale.

#### 2.2.4 Measure of intrinsic motivation

Intrinsic motivation was measured using the Intrinsic Goal Orientation (IGO) subscale of the Motivated Strategies for Learning Questionnaire (MSLQ; Pintrich, 1991). The subscale contains four items rated on a five-point Likert scale and assesses the extent to which students engage in learning tasks for challenge, curiosity, or mastery.

#### 2.2.5 Measure of self-efficacy

The General Academic Self-Efficacy Scale (GASE; Nielsen et al., 2018) was used to assess general academic self-efficacy among higher education students using a five-point response scale. The four items measured the respondents' beliefs about their ability to achieve goals in an academic environment.

### 2.3 Procedure

Data were collected between September and October 2025, using an online platform. Participants were recruited using convenience sampling (e.g., in classes and via acquaintances). Participants were informed that the survey was anonymous and that completion implied consent for scientific use. On average, completing the questionnaire took approximately 35 min. All procedures complied with the ethical standards of the institutional and national research committees and the 1975 Declaration of Helsinki, as revised in 2000. The study was approved as part of an ongoing project by the ethics committee (UKF/370/2025/191013:024).

### 2.4 Data analysis

Descriptive statistics and correlation analyses for all measured variables were conducted using Jeffreys's Amazing Statistics Program (JASP, version 0.19.3). Pearson's correlation coefficient was used for all variables whose distributions met the recommended thresholds for approximate normality (skewness and kurtosis within ±1; Kline, 2015). Spearman's correlation coefficient was applied for the GPA AS variable, as it was not normally distributed (skewness = 1.11, kurtosis = 2.48). Assumptions of linearity, homoscedasticity, and the absence of multivariate outliers were inspected, and no substantial violations were detected. The GPA AS variable was coded such that lower values (closer to 1.00) indicated higher academic success; therefore, negative correlations reflected better student performance. Sequential mediation analyses were performed using Jamovi 2.6.44.0. The analyses employed a regression-based general linear model (GLM) mediation model. To address the potential non-normality of the sampling distribution of indirect effects, a bias-corrected bootstrapping procedure was applied (Hayes, 2018). For each mediation model, 5,000 bootstrap samples were generated, and the statistical significance of the indirect effects was evaluated based on 95% bootstrap confidence intervals. Indirect effects were considered statistically significant only if the confidence interval did not include zero (Hayes, 2018). Four sets of mediation pathways were tested to examine the effects of critical thinking on academic success through academic control, self-efficacy, and intrinsic motivation. Effect sizes (Pearson's *r*) were interpreted according to Cohen's (1988) conventional thresholds: ~0.10 small, ~0.30 medium, and ~0.50 large.

## 3 Results

### 3.1 Descriptive statistics and correlations

Descriptive statistics (M, SD), internal consistency coefficients (Cronbach's α and McDonald's ω), and correlations between the measured variables are reported in Table 1. The CT skills scale demonstrated lower reliability (α = 0.51), which is typical for brief ability-based measures, particularly given the small number of items (9) and dichotomous format; such lower values are considered acceptable in this context (Izah et al., 2023). Most variables correlated weakly to moderately with each other (*r* = ±0.13 to ±0.53; *p* < 0.05). No significant associations were observed between GPA and CT disposition or between CT skills and self-efficacy. The CT disposition dimensions—Critical Openness (CO) and Reflective Skepticism (RS)—were strongly correlated with each other (*r* = 0.58^***^) as well as with the total score (r_CO = 0.92^***^ and r_RS = 0.86^***^). For this reason, and for model parsimony, subsequent analyses were conducted using only the total score. For completeness, the analyses were also recalculated with the individual CO and RS dimensions, yielding very similar results with only minor deviations.

**Table 1:** Descriptive statistics and correlations.

Variable		*N*	M	SD	α	ω	1	2	3	4	5	6	7	8	9
Age		263	21.74	1.56											
Gender	Female	213													
	Male	50													
Study level	Undergraduate	199													
	Graduate	64													
1 CT dispositions		263	44.14	5.67	0.79	0.80	—								
2 CTD—CO		263	28.25	3.61	0.68	0.69	0.92^***^	—							
3 CTD—RS		263	15.89	2.77	0.72	0.73	0.86^***^	0.58^***^	—						
4 CT skills		263	6.69	1.75	0.51	0.52	0.15^*^	0.18^**^	0.08	—					
5 GPA		253	1.72	0.47	—	—	−0.09	−0.03	−0.12	−0.26^**^	—				
6 Perceived AS		263	12.32	1.86	0.78	0.80	0.30^***^	0.26^***^	0.28^***^	0.16^*^	−0.30^***^	—			
7 Academic control		263	29.80	5.08	0.74	0.75	0.39^***^	0.35^***^	0.34^***^	0.30^***^	−0.35^***^	0.58^***^	—		
8 Self-efficacy		263	14.43	2.83	0.65	0.65	0.36^***^	0.32^***^	0.32^***^	0.11	−0.21^***^	0.51^***^	0.43^***^	—	
9 Intrinsic mot.		263	16.34	4.56	0.69	0.70	0.37^***^	0.36^***^	0.29^***^	0.21^***^	−0.13^*^	0.41^***^	0.35^***^	0.38^***^	—

^*^p < 0.05, ^**^p < 0.01, ^***^p < 0.001.

CT, critical thinking; CO, critical openness; RS, reflective skepticism; GPA, grade point average; AS, academic success; mot., motivation.

### 3.2 The sequential mediation path models

**Figure 5:**
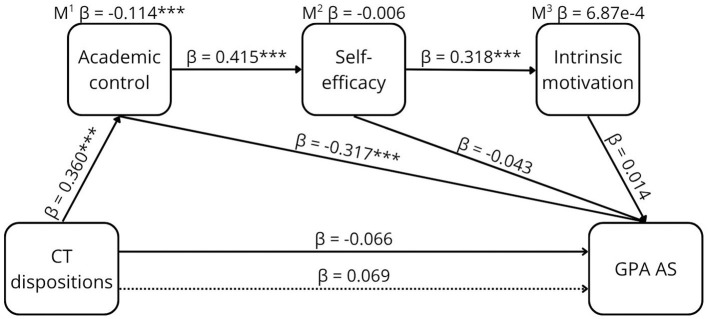
The relationship between CTD and GPA AS. *p < 0.05, ***p < 0.001, β = standardized estimate. M^1^ mediation path CTD ⇒ AC ⇒ GPA AS. M^2^ mediation path CTD ⇒ AC ⇒ SEF ⇒ GPA AS. M^3^ mediation path CTD ⇒ AC ⇒ SEF ⇒ MOT ⇒ GPA AS. → Total effect, -- → Direct effect.

**Figure 6:**
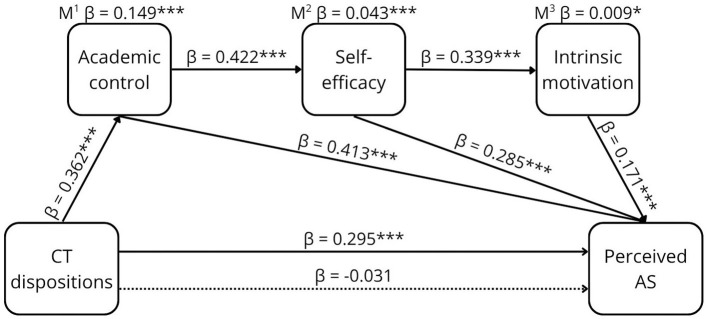
The relationship between CTD and perceived AS. *p < 0.05, ***p < 0.001, β =standardized estimate. M^1^ mediation path CTD ⇒ AC ⇒ perceived AS. M^2^ mediation path CTD ⇒ AC ⇒ SEF ⇒ perceived AS. M^3^ mediation path CTD ⇒ AC ⇒ SEF ⇒ MOT ⇒ perceived AS. → Total effect, -- → Direct effect.

**Figure 7:**
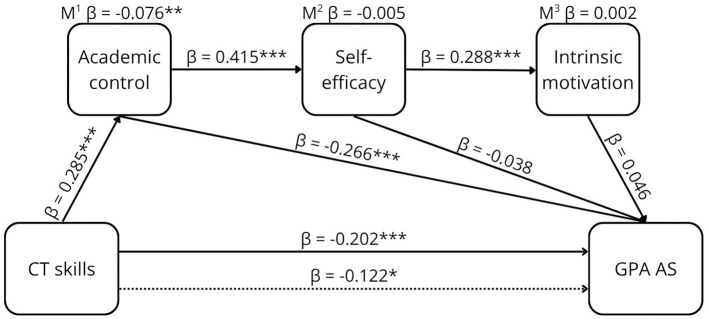
The relationship between CT skills and GPA AS. *p < 0.05, **p < 0.01, ***p < 0.001, β =standardized estimate. M^1^ mediation path CTS ⇒ AC ⇒ GPA AS. M^2^ mediation path CTS ⇒ AC ⇒ SEF ⇒ GPA AS. M^3^ mediation path CTS ⇒ AC ⇒ SEF ⇒ MOT ⇒ GPA AS. → Total effect, -- → Direct effect.

**Figure 8:**
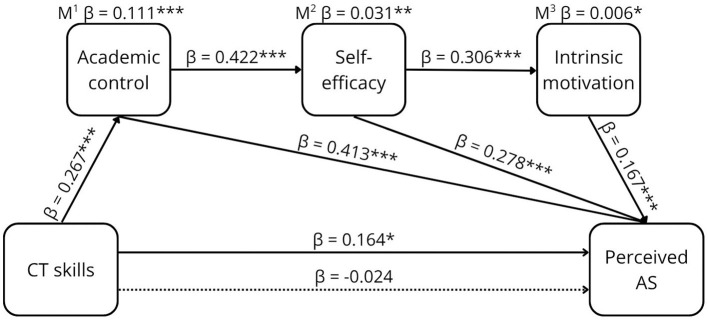
The relationship between CT skills and perceived AS. *p < 0.05, **p < 0.01, ***p < 0.001, β =standardized estimate. M^1^ mediation path CTS ⇒ AC ⇒ perceived AS. M^2^ mediation path CTS ⇒ AC ⇒ SEF ⇒ perceived AS. M^3^ mediation path CTS ⇒ AC ⇒ SEF ⇒ MOT ⇒ perceived AS. → Total effect, -- → Direct effect.

The results of the sequential mediation are shown in Table 2 and graphically presented in Figures 5–8.

**Table 2:** Estimates for the three-mediator sequential models.

Type	*N*	Predictor	Mediators	Dependent	Estimate/B	SE	95% C.I. (a)		β
							Lower	Upper	
Indirect	253	CTD	AC	GPA AS	−0.009	0.009	−0.017	−0.005	−0.114^***^
			AC ⇒ SEF		−5.32e-4	8.48e-4	−0.002	0.001	−0.006
			AC ⇒ SEF ⇒ MOT		5.63e-5	2.53e-4	−5.17e-4	6.52e-4	6.87e-4
Direct					0.006	0.005	−0.004	0.0161	0.069
Total					0.005	0.005	−0.015	0.004	0.066
Indirect	263	CTD	AC	Perceived AS	0.059	0.012	0.036	0.088	0.149^***^
			AC ⇒ SEF		0.017	0.005	0.009	0.030	0.043^***^
			AC ⇒ SEF ⇒ MOT		0.003	0.001	0.002	0.008	0.009^**^
Direct					−0.012	0.019	−0.056	0.029	−0.031
Total					0.117	0.023	0.070	0.164	0.295^***^
Indirect	253	CTS	AC	GPA AS	−0.020	0.007	−0.037	−0.009	−0.076^**^
			AC ⇒ SEF		−0.001	0.002	−0.006	0.003	−0.005
			AC ⇒ SEF ⇒ MOT		4.22e-4	6.07e-4	−6.98e-4	0.002	0.002
Direct					−0.033	0.017	−0.065	−0.002	−0.122^*^
Total					−0.054	0.016	−0.083	−0.023	−0.202^***^
Indirect	263	CTS	AC	Perceived AS	0.142	0.036	0.084	0.218	0.111^***^
			AC ⇒ SEF		0.040	0.013	0.021	0.073	0.031^**^
			AC ⇒ SEF ⇒ MOT		0.007	0.003	0.002	0.017	0.006^*^
Direct					−0.031	0.061	−0.162	0.102	−0.024
Total					0.218	0.078	0.056	0.361	0.164^*^

^*^p < 0.05, ^**^p < 0.01, ^***^p < 0.001.

AC, academic control; SEF, self-efficacy; MOT, motivation; C.I. (a), Adjusted Bias-Corrected Bootstrap Confidence Interval; SE, Standard Error; β, standardized estimate.

#### 3.2.1 Model CT dispositions → GPA AS

The total effect of CT disposition on GPA AS was not significant (β = 0.066, [95% CI −0.015, 0.004]). Sequential mediation through academic control, self-efficacy, and intrinsic motivation was not confirmed (β = 5.63e-5 [95% CI −5.17e-4, 6.52e-4]). The only significant indirect effect was mediation via academic control (β = −0.114, [95% CI −0.017, −0.005]), indicating a weak effect. After accounting for the mediators, the direct effect of CT dispositions on GPA AS was not observed, suggesting full mediation through academic control.

#### 3.2.2 Model CT dispositions → perceived AS

CT disposition significantly and positively predicted perceived AS, with a medium effect size (β = 0.295, [95% CI 0.070, 0.163]). The total sequential mediation through the three mediators was significant but weak (β = 0.009, [95% CI 0.001, 0.009]). After including the mediators, the direct effect of CT disposition on perceived AS was not significant, indicating indirect-only mediation.

#### 3.2.3 Model CT skills → GPA AS

CT skills showed a weak negative prediction of GPA AS (β = −0.202, [95% CI −0.083, −0.023]). Mediation through all three mediators was not significant (β = 0.002, [95% CI −6.98e-4, 0.002]). Mediation via academic control was significant, with a weak effect (β = −0.076, [95% CI −0.037, −0.009]). Even after accounting for the mediator, a weak but significant effect of CT skills on GPA AS remained (β = −0.122, [95% CI −0.065, −0.002]), indicating partial mediation.

#### 3.2.4 Model CT skills → perceived AS

CT skills were a weak positive predictor of perceived AS (β = 0.164, [95% CI 0.056, 0.361]). The sequential mediation through the three mediators was significant with a weak effect (β = 0.006, [95% CI 0.002, 0.017]). The direct effect did not remain after mediators were included, indicating full mediation.

### 3.3 Predictive effects of the three mediators

In all mediation models, the positive effect of academic control on self-efficacy was confirmed, with the strongest effect observed in the relationship between CT skills and GPA AS (β = 0.422, [95% CI 0.182, 0.294]). Likewise, the subsequent positive effect of self-efficacy on intrinsic motivation was consistent and was observed to be strongest in the relationship between CT disposition and perceived AS (β = 0.339, [95% CI 0.371, 0.736]).

## 4 Discussion

This study aimed to examine the relationship between CT and AS (H1a–H1d) through three sequentially arranged mediators. We hypothesized that academic control influences self-efficacy, which subsequently affects intrinsic motivation. These mediators were analyzed in the following order across four models: between CT dispositions and GPA AS (H2–H4), CT dispositions and perceived AS (H5–H7), CT skills and GPA AS (H8–H10), and finally CT skills and perceived AS (H11–H13). Full mediation, including all three mediators, was confirmed for both CT skills and dispositions, but only for perceived AS. GPA AS was directly predicted only by CT skills, whereas CT dispositions influenced GPA only indirectly via academic control.

The hypothesized sequential mediation model was confirmed only for perceived AS. This construct of academic success covers a wide range of psychosocial aspects of student functioning. In the examined models, the relationship between CT and perceived AS was mediated by three variables—academic control, self-efficacy, and intrinsic motivation—with academic control showing the strongest effect. The literature consistently indicates that each of these variables significantly influences academic satisfaction (Khaleghinezhad et al., 2016; Doménech-Betoret et al., 2017; Ribeiro et al., 2022). Higher internal control and self-efficacy may promote a more interactive study environment (Chan et al., 2009), increase learning engagement (Miao et al., 2025), and facilitate adaptation to higher education (Auliya et al., 2023). Higher values of these interpersonal variables are associated with greater intrinsic motivation driven by personal interest rather than external rewards (Artino, 2012; Torres et al., 2025). According to (Sagone and De Caroli 2014), these variables shape students' overall self-concept—the more students believe in their control and efficacy in the academic environment, the more positively they perceive themselves in the present and future. These concepts manifest primarily in students' feelings about their studies. In our models, the indirect path of these three mediators was observed in every analysis, providing empirical support for Bandura's social learning theory (1986). These constructs are closely interconnected (Elliott and Del Puerto, 2014; Graham, 2007) and mutually influence each other (Heidari et al., 2023), confirming that academic experience results from a complex psychological chain rather than a single isolated factor.

Developed CT dispositions or skills may foster students' belief in control over their academic outcomes, strengthening confidence in their abilities to successfully manage academic tasks, and potentially increasing motivation toward academic activities. These psychological resources may contribute to positive academic experiences. Since the effect of CT on perceived AS disappears after controlling for mediators, we can conclude that it is influenced not directly by critical thinking processes but through multiple social cognitive mechanisms. This aligns with a longitudinal study showing that academic self-concept positively affects perceptions of the learning environment (Guo et al., 2022).

The mediation model, including the performance component—GPA AS—was confirmed only with academic control as a mediator. Our findings further showed that GPA AS is directly predicted only by CT skills and indirectly predicted through academic control by both CT skills and dispositions. This highlights the central role of academic control, which links both CT skills and dispositions to GPA AS. According to Bandura's social learning theory (1986), locus of control initiates many motivational and behavioral processes and may thus be a key factor in AS. Students' beliefs in their control over academic outcomes significantly affect multiple supportive factors of academic functioning, including reduced procrastination, more active study approaches, better self-regulation, persistence, and overall engagement (Al Mulhim, 2021; Muca et al., 2023; Rustamov et al., 2024; Torres et al., 2025). A possible explanation is also offered by the Reflective Judgment Model (King and Kitchener, 2004), which describes the stages of CT. The pre-reflective stage involves the belief that knowledge is certain, unambiguous, and comes from external authorities, thus reducing the need for a personal critical evaluation of evidence. This mode of thinking aligns with an external locus of control, as both shift the source of truth and responsibility for outcomes outside the individual. Hence, attribution processes may be the primary mechanism through which CT influences students' academic success. GPA AS may also be influenced by various external factors, such as teacher characteristics, course difficulty, or class size (Yeritsyan et al., 2022; Shin et al., 2025), which highlights the importance of individual differences in responses to these conditions. Attribution processes, such as academic control, may therefore exert a stronger influence than motivation or self-efficacy. Consequently, the positive effect of CT skills on GPA AS (Orhan, 2022) can be reinforced by internal academic control (Flor et al., 2013), which determines whether and how students work with external factors, adapt learning strategies, and compensate for academic demands. Similarly, higher CT dispositions may support internal academic control (Stupnisky et al., 2008), leading students to perceive academic outcomes because of their efforts, thereby increasing engagement. Unlike CT skills, the effect of CT dispositions on GPA AS is likely mediated only through academic control and not directly. The observed differences in the effects of social cognitive variables on perceived and GPA-based academic success may be explained by their underlying nature. Self-efficacy and intrinsic motivation reflect students' subjective beliefs about their capabilities and engagement and are therefore more closely aligned with perceived academic success as a psychosocial evaluation of academic functioning. In contrast, GPA reflects an objective, performance-based outcome that is shaped not only by individual characteristics but also by external factors. In this context, the influence of subjective characteristics such as self-efficacy and motivation may be attenuated, while academic control—representing a more behaviorally relevant orientation toward managing academic demands—remains a key factor in how students respond to these conditions. Additionally, the self-reported nature of perceived academic success may contribute to its stronger association with self-reported social cognitive variables.

The present findings also point to the complexity of critical thinking and support Facione's (2000) original distinction between the two components of CT: dispositions, understood as personality traits and predispositions toward CT, and skills, understood as actual performance and the ability to solve cognitive tasks. We demonstrated that both components contribute to academic success to varying degrees via different mechanisms. CT skills were more strongly related to GPA AS than to perceived AS. This finding aligns with previous studies suggesting that GPA is more strongly linked to CT skills than dispositions (Dissen, 2023; Orhan, 2022). CT skills and GPA are both objectively measured performance variables and are relevant in examination situations, which explains their close relationship. In contrast, dispositions showed stronger relationships with perceived AS but did not directly predict GPA AS, consistent with prior research (Whitney et al., 2016; Partido and Soto, 2019; Lukačková and Šeboková, 2024). These studies suggest that AS in relation to CT dispositions should be measured differently from GPA. The variable perceived AS, for which both CT components are relevant, serves this purpose. Previous studies have shown that CT is related to better communication (Heo, 2015), knowledge management (Indrašiene et al., 2021), and emotional intelligence skills (Akbarilakeh et al., 2018). Therefore, CT dispositions (Yüce, 2023) or skills (Huamán-Tapia et al., 2023) may help students experience more positive emotions in relation to studying, communicate more effectively, organize study tasks better, and generally feel more satisfied with their academic environment.

The present findings further underscore the distinct yet complementary roles of critical thinking skills and dispositions within the academic context. Whereas, skills primarily support performance in structured cognitive tasks, dispositions appear to fulfill a regulatory and activation function, determining whether and when such skills are enacted. In line with Halpern (1998), students may demonstrate proficiency in analytical strategies within well-defined academic demands yet fail to transfer these skills to novel or ill-structured situations in the absence of appropriate dispositions. Given that higher education is expected to prepare students for the complexities of the labor market, these findings highlight the importance of fostering both critical thinking skills and dispositions in an integrated manner.

### 4.1 Limitations and future research directions

One major limitation of the present study is its cross-sectional design, which does not allow causal inferences. Although mediation was tested, it represents only statistical modeling of hypothetical causal relationships and not proof of actual causality. The literature also suggests the potential reverse effects, highlighting the need for longitudinal studies to track the development of variables over time. Another limitation is the measurement of AS via GPA, which students self-reported using institutional records. Future research should use official records when available. Additionally, the WGCTA used to measure CT skills demonstrated relatively low internal consistency (α = 0.51). Although lower reliability is more common in performance-based measures with dichotomous items, this limitation should be considered when interpreting the findings. The effect sizes in this study were small for some significant relationships. However, given the multifactorial nature of the studied variables, small effects were expected and did not compromise the model's fit.

### 4.2 Theoretical and practical implications

These findings have important implications for the study of academic success by highlighting its complexity and questioning the sufficiency of GPA as the sole indicator. The results support the distinction between performance-based (GPA AS) and psychosocial (perceived AS) components, which remains relatively underemphasized in the current literature. Importantly, these findings suggest that academic success cannot be effectively addressed through uniform approaches, as its distinct components are shaped by different psychological mechanisms. This differentiation opens up space for more targeted pedagogical interventions that separately address performance outcomes and students' subjective academic experience.

This study also offers a more nuanced perspective on critical thinking by demonstrating the distinct roles of CT disposition and skills in relation to academic success. While CT skills appear to be more directly linked to performance outcomes, CT dispositions operate indirectly, primarily through social cognitive mechanisms. This distinction has practical implications, as it suggests that the development of critical thinking in higher education should not be limited to cognitive skill training but should also include interventions aimed at fostering students' willingness to engage in critical thinking, such as promoting epistemic curiosity, openness to alternative perspectives, and reflective judgment.

Furthermore, these findings contribute to a deeper understanding of the psychological processes underlying students' success and retention in higher education. Academic control emerges as a key factor for GPA AS, highlighting its role as a central mechanism through which both CT skills and dispositions translate into performance outcomes. This suggests that interventions aimed at strengthening students' internal attributions of success and failure may be particularly effective in improving their academic performance. In contrast, perceived AS is shaped by self-efficacy and intrinsic motivation, indicating that students' subjective academic experience is influenced by a broader network of interrelated socio-cognitive factors. From a practical perspective, this implies that supporting student retention and wellbeing requires not only fostering cognitive competencies but also systematically strengthening students' sense of competence, agency, and intrinsic engagement with learning.

## Conclusion

This study examined the association between CT and AS mediated by three social cognitive factors. The findings show that CT skills and dispositions relate to AS differently, depending on whether it is operationalized objectively or subjectively. Subjective AS is shaped by multiple factors—particularly academic control, self-efficacy, and motivation—whereas objective AS is primarily mediated by academic control. As CT is widely understood as a multidimensional construct comprising skills and dispositions, this study highlights the multidimensional structure of AS, which is often treated as unidimensional. Accordingly, future research should assess both CT and AS in a comprehensive manner that reflects their internal differentiation.

## Data Availability

The raw data supporting the conclusions of this article will be made available by the authors, without undue reservation.
