# Supplementary material for: Enhancing academic success in higher education: the predictive role of critical thinking through sequential mediation of academic control, self-efficacy, and intrinsic motivation
Source: Front Psychol. 2026 May 19;17:1783512. doi: 10.3389/fpsyg.2026.1783512 (PMC13227591; doi:10.3389/fpsyg.2026.1783512)
Supplement: Supplementary file 1 [file Table_1.docx]

Apendix A

| Path | Source |
| --- | --- |
| CT dispositions ***⇒*** GPA AS | Although this relationship has been less extensively studied compared to skills, meta-analyses consistently report a positive association between CT dispositions and GPA (Ross et al., 2013: *r* = 0.21; Fong et al., 2017: *r* = 0.31; Orhan, 2022: *r* = 0.35). |
| CT dispositions ***⇒*** perceived AS | Perceived academic success represents a less frequently examined component; however, the literature provides empirical support for its positive relationship with CT dispositions through conceptually related constructs, such as satisfaction with research courses (Landa-Blanco & Cortés-Ramos, 2021), interest in learning (Tong et al., 2023), and academic grit (Yüce, 2023). Higher levels of critical thinking dispositions have also been shown to predict greater major satisfaction (Eom, 2019), all with small to moderate strength of the relationship. |
| CT skills ***⇒*** GPA AS | The relationship between CT skills and GPA is well established in the literature and supported by meta-analytic evidence (Ross et al., 2013: *r* = 0.33; Fong et al., 2017: *r* = 0.25; Orhan, 2022: *r* = 0.47). |
| CT skills – perceived AS | Compared to dispositions, the association between CT skills and perceived academic success has received less attention; nevertheless, empirical evidence suggests positive relationships with related constructs, such as student satisfaction (Al-Zarfi et al., 2024), as well as findings indicating that critical thinking skills predict satisfaction with studies and overall academic experience (Huamán-Tapia et al., 2023; Shcheglova et al., 2024) also with small to moderate strenght. |
| CT dispositions ***⇒*** academic control | Research on CT and academic control has mainly focused on skills. Studies on dispositions often suggest the opposite direction, with locus of control predicting CT dispositions (Tahrir et al., 2020; Oguz & Sariçam, 2016), although longitudinal evidence points to a reciprocal relationship, with dispositions also predicting academic control (Stupnisky et al., 2008). |
| CT dispositions ***⇒*** self-efficacy | The relationship between CT dispositions and self-efficacy is relatively well established, with consistent evidence of a moderate positive association. Although self-efficacy is often treated as a predictor of CT dispositions (Taşğın & Dilek, 2023), evidence suggests that the relationship may be reciprocal, with support also for the reverse directional association (Çelikkol & Konik, 2023; Heidari et al., 2023; Yurt & Hayli, 2025). |
| CT dispositions ***⇒*** motivation | The relationship between motivation and CT dispositions is well supported in the empirical literature and may be reciprocal, with evidence suggesting that motivation predicts CT dispositions (Jaramillo Gómez et al., 2025), while CT dispositions may also predict motivation (Kuloğlu & Orhan, 2024). |
| CT skills ***⇒*** academic control | There is substantial empirical support for a positive relationship between critical thinking skills and academic control; reciprocity also appears to characterize this relationship, as empirical evidence indicates that academic control can act as a predictor of CT skills (Tambunan, 2021), while CT skills may also predict academic control (Flor et al., 2013). |
| CT skills ***⇒*** self-efficacy | Self-efficacy shows a well-established positive relationship with CT skills. This relationship also appears to be reciprocal, as empirical evidence indicates that self-efficacy can act as a predictor of CT skills (Arif et al., 2025), while CT skills may also predict self-efficacy (Heidari Gorji et al., 2018). |
| CT skills ***⇒*** motivation | Similarly to dispositions, the relationship between motivation and CT skills is well supported in the literature and appears to be reciprocal, with evidence suggesting that motivation predicts CT skills (Jaramillo Gómez et al., 2025), while skills also predict motivation (Agoes Salim et al., 2024; Points III, 2003). |
| academic control ***⇒*** GPA AS | Findings on this relationship are extensive. Meta-analytic evidence shows that academic control predicts academic achievement directly (Richardson et al., 2012), although other meta-analysis suggests an indirect effect via self-efficacy and motivation (Haidari et al., 2023). More recent research, however, again supports a direct effect (Althubaiti et al., 2025). |
| self-efficacy ***⇒*** GPA AS | Meta-analytic evidence shows that self-efficacy predicts academic achievement directly and indirectly through motivation (Haidari et al., 2023; Qi et al., 2024). |
| motivation ***⇒*** GPA AS | Similarly, motivation is a consistent predictor of academic achievement, as supported by meta-analytic evidence (Qi et al., 2024), while longitudinal meta-analytic findings suggest that this relationship may be reciprocal (Vu et al., 2024). |
| academic control ***⇒*** perceived AS | Research on perceived AS is more limited compared to GPA-based outcomes; however, existing evidence suggests that academic control predicts perceived stress and student satisfaction (Dumitrescu, 2016), school satisfaction (Rustamov et al., 2024), and overall life satisfaction (Khaleghinezhad et al., 2016). |
| self-efficacy ***⇒*** perceived AS | There is substantial empirical support for self-efficacy as a predictor of academic engagement, with meta-analytic findings indicating a moderate effect (Fatimah et al., 2024). Additionally, self-efficacy has been shown to predict perceived stress and student satisfaction (Chahal et al., 2025; Dumitrescu, 2016), as well as subjective academic success (Alzukari, 2024). |
| motivation ***⇒*** perceived AS | Similarly, it has been shown that motivation predicts perceived AS, including subjective academic success (Alzukari, 2024), student satisfaction (Chahal et al., 2025), and students’ experience in the academic environment (Kasemy et al., 2022). |
| academic control ***⇒*** self-efficacy | Meta-analytic evidence examining all three constructs indicates that academic motivation is predicted by both academic control and self-efficacy, while academic control does not predict self-efficacy (Haidari et al., 2023). However, other studies have reported a positive effect of locus of control on self-efficacy (Anderson et al., 2005; Elliott & Del Puerto, 2014; Papoulidi & Maniadaki, 2025; Uzun & Karataş, 2020). Additional empirical evidence also supports the role of both academic control (Anderson et al., 2005; Li et al., 2024) and self-efficacy (Ahmadi et al., 2023; Chen, 2024; Qi et al., 2024) as predictors of academic motivation. |
| academic control ***⇒*** motivation |  |
| self-efficacy ***⇒*** motivation |  |

References

Ahmadi, A., Ziapour, A., Lebni, J. Y., & Mehedi, N. (2023). Prediction of academic motivation based on variables of personality traits, academic self-efficacy, academic alienation and social support in paramedical students. *Community health equity research & policy*, *43*(2), 195-201. [10.1177/0272684X211004948](https://doi.org/10.1177/0272684X211004948)

Agoes Salim, R. M., Felicia, M. S., & Setiamurti, A. (2024). The Missing Link: Academic Motivation as a Mediator in the Relationship between Mindfulness and Critical Thinking Skills in Indonesian First-year Undergraduates. *The Open Psychology Journal*, *17*(1). [10.2174/0118743501342275241204113406](http://dx.doi.org/10.2174/0118743501342275241204113406)

Althubaiti, S.M., Alharbi, N.S., Althubaiti, A. Amal Alzahrani & Sajida Agha (2025) Locus of Control, Learning Styles, and Academic Achievement of Saudi Pre-professional Medical Students: A Cross-sectional Study. *Acad Psychiatry* **49**, 65–69. <https://doi.org/10.1007/s40596-024-02056-9>

Alzukari, R.,, "Academic self-efficacy, achievement motivation, and academic success of international graduate students" (2024). *Theses and Dissertations*. 6313.
https://scholarsjunction.msstate.edu/td/6313

Al-Zarfi, A. J. K., Qalavandi, H., & Hosni, M. (2024). The Effect of E-Learning on Academic Progress, Critical Thinking, Higher Thinking and Student Satisfaction with the Mediating Role of Academic Self-Efficacy, Learning Motivation and Student Participation. *Kurdish Studies*, *12*(1), 4935-4951. <https://doi.org/10.58262/ks.v12i1.358>

Anderson, A., Hattie, J., & Hamilton, R. J. (2005). Locus of Control, Self‐Efficacy, and Motivation in Different Schools: Is moderation the key to success?. *Educational psychology*, *25*(5), 517-535. 10.1080/01443410500046754

Arif, S., Hariani, L. S., & Brihandhono, A. (2025). E-Learning, Self-Efficacy, and Motivation: Their Influence on Critical Thinking in IPAS Learning. *Journal of General Education and Humanities*, *4*(4), 1669–1678. <https://doi.org/10.58421/gehu.v4i4.737>

Çelikkol, A. K., & Konik, A. K. (2023). The Role of Critical Thinking Dispositions and Depressive Symptoms in Predicting Teacher Candidates' Perceptions of" Teacher Self-Efficacy". *African Educational Research Journal*, *11*(3), 293-299. <https://doi.org/10.30918/AERJ.113.23.047>

Elliott, J. W., & Del Puerto, C. L. (2014). Self-efficacy, motivation, and locus of control among male and female construction management students. In *2014 ASEE Annual Conference & Exposition* (pp. 24-1077). https://peer.asee.org/23010

Chahal A, Kadian R, Yadav R, Prakash C (2026), "Self-efficacy, learning motivation and academic satisfaction of university students: mediating role of classroom engagement". *Journal of Applied Research in Higher Education*, Vol. 18 No. 1 pp. 238–253, doi: <https://doi.org/10.1108/JARHE-06-2024-0305>

Chen, L. (2024). Delving into the role of self-efficacy in predicting motivation and engagement among music learners. *Learning and Motivation*, *86*, 101961. <https://doi.org/10.1016/j.lmot.2024.101961>

Dumitrescu, G. A. (2016). Self-efficacy, locus of control, perceived stress and student satisfaction as correlates of dissertation completion. *Andrews University.*

Eom, S., Choi, Y. J., Lee, B. H. (2019). The influence of problem-solving ability and critical thinking on the major satisfaction of dental hygiene student-focused on mediating effects of critical Thinking. *Journal of the Korea Convergence Society*, *10*(12), 151-158. <https://doi.org/10.15207/JKCS.2019.10.12.151>

Fatimah, S., Murwani, F. D., Farida, I. A., & Hitipeuw, I. (2024). Academic self-efficacy and its effect on academic engagement: Meta-analysis. International Journal of Instruction, 17(1), 271-294. https://doi.org/10.29333/iji.2024.17115a

Flor, R. K., Bita, A., Monir, K. C., Zohreh, Z. Z. (2013). The effect of teaching critical and creative thinking skills on the locus of control and psychological well-being in adolescents. *Procedia-Social and Behavioral Sciences*, *82*, 51-56.

Fong, C. J., Kim, Y., Davis, C. W., Hoang, T., & Kim, Y. W. (2017). A meta-analysis on critical thinking and community college student achievement. *Thinking Skills and Creativity*, *26*, 71-83. [https://doi.org/10.1016/j.tsc.2017.06.002](https://psycnet.apa.org/doi/10.1016/j.tsc.2017.06.002)

Haidari S. M., Koçoğlu A., Kanadlı S. (2023) ‘Contribution of Locus of Control, Self-Efficacy, and Motivation to Student Achievement: A Meta-Analytic Structural Equation Modelling’, *Journal on Efficiency and Responsibility in Education and Science,* vol. 16, no. 3, pp. 245-261. <http://dx.doi.org/10.7160/eriesj.2023.160308>

Heidari, S., Gheisari, Z., Hashemi, Z. (2023). The Relationship between Critical Thinking and Academic Self-Efficacy: The Moderating Role of Self-Directed Learning. Biquarterly Journal of Cognitive Strategies in Learning, 11(20), 47-66. [10.22084/j.psychogy.2022.25612.2425](https://doi.org/10.22084/j.psychogy.2022.25612.2425)

Heidari Gorji A M, Shafizad M, Soleimani A, Darabinia M, Goudarzian A H. (2018) Path Analysis of Self-Efficacy, Critical Thinking Skills and Emotional Intelligence for Mental Health of Medical Students.Iran J Psychiatry Behav Sci.2018;12(4):e59487.<https://doi.org/10.5812/ijpbs.59487>.

Huamán-Tapia, E., Almanza-Cabe, R. B., Sairitupa-Sanchez, L. Z., Morales-García, S. B., Rivera-Lozada, O., Flores-Paredes, A., & Morales-García, W. C. (2023). Critical Thinking, Generalized Anxiety in Satisfaction with Studies: The Mediating Role of Academic Self-Efficacy in Medical Students. *Behavioral Sciences*, *13*(8), 665. <https://doi.org/10.3390/bs13080665>

Jaramillo Gómez, D. L., Álvarez Maestre, A. J., Parada Trujillo, A. E., Pérez Fuentes, C. A., Bedoya Ortiz, D. H., & Sanabria Alarcón, R. K. (2025). Determining Factors for the Development of Critical Thinking in Higher Education. *Journal of Intelligence*, *13*(6), 59. <https://doi.org/10.3390/jintelligence13060059>

Kasemy, Z. A., Kabbash, I., Desouky, D., El-Raouf, S. A., Aloshari, S., & El Sheikh, G. (2022). Perception of educational environment with an assessment of motivational learning strategies and emotional intelligence as factors affecting medical students' academic achievement. *Journal of education and health promotion*, *11*, 303. <https://doi.org/10.4103/jehp.jehp_1772_21>

Khaleghinezhad, S. A., Shabani, M., Hakimzadeh, R., Nazari Shaker, H., Amerian, M. (2016). Prediction of high school students’ life satisfaction and academic performance based on locus of control and self-esteem. *International journal of school health*, *3*(3), 1-7. [10.17795/intjsh-31924](https://doi.org/10.17795/intjsh-31924)

Kuloğlu, A. & Orhan, F.G. (2024). The Relationships Between Self-Regulation, Critical Thinking Dispositions, and Academic Motivation Among Secondary School Students: A Structural Equation Modeling, International Journal of Eurasian Education and Culture, 9(28), 538-554 <http://dx.doi.org/10.35826/ijoecc.2849>

Landa-Blanco, M., Cortés-Ramos, A. (2021). Psychology students' attitudes towards research: the role of critical thinking, epistemic orientation, and satisfaction with research courses. *Heliyon*, *7*(12). <https://doi.org/10.1016/j.heliyon.2021.e08504>

Li, X., Mao, Z., Zhao, J., Wang, Y., & Wang, Y. (2024). Relationships among locus of control, academic engagement, and achievement motivation in Chinese adolescents. *Social Behavior and Personality: an international journal*, *52*(5), 12640E-12655E.

Oguz, A., Sariçam, H. (2016). The Relationship between Critical Thinking Disposition and Locus of Control in Pre-Service Teachers. *Journal of Education and Training Studies*, *4*(2), 182-192.

Orhan, A. (2022). The relationship between critical thinking and academic achievement: A meta-analysis study. *Psycho-Educational Research Reviews*, *11*(1), 283-299. <https://doi.org/10.52963/PERR_Biruni_V11.N1.18>

Papoulidi, A., & Maniadaki, K. (2025). The Mediating Role of Self-Efficacy in the Relationship Between Locus of Control and Resilience in Primary School Students. *European Journal of Investigation in Health, Psychology and Education*, *15*(7), 138. https://doi.org/10.3390/ejihpe15070138

Points III, G. L. (2024). Critical thinking and intrinsic motivation in secondary science. *University of North Carolina Wilmington* <https://hdl.handle.net/20.500.14481/944>

Qi, B., Ma, L., & Wang, X. (2024). Using meta-analytic path analysis to examine mechanisms relating students’ perceived feedback, motivation, self-efficacy, and academic performance. *Learning and Motivation*, *88*, 102059

Tahrir, T., Nurdin, F. S., Damayanti, I. R. (2020). The role of critical thinking as a mediator variable in the effect of internal locus of control on moral disengagement. International Journal of Instruction, 13(1), 17-34. <https://doi.org/10.29333/iji.2020.1312a>

Tambunan, L. (2021). Implementasi Pembelajaran Cooperative Learning dan Locus of Control dalam Meningkatkan Kemampuan Berpikir Kritis. *Jurnal Cendekia : Jurnal Pendidikan Matematika*, *5*(2), 1051-1061. <https://doi.org/10.31004/cendekia.v5i2.491>

Tong, L. K., Au, M. L., Li, Y. Y., Ng, W. I., Wang, S. C. (2023). The mediating effect of critical thinking between interest in learning and caring among nursing students: a cross-sectional study. *BMC nursing*, *22*(1), 30.

Richardson, M., Abraham, C., & Bond, R. (2012). Psychological correlates of university students' academic performance: a systematic review and meta-analysis. *Psychological bulletin*, *138*(2), 353.

Ross, D., Loeffler, K., Schipper, S., Vandermeer, B., Allan, G. M. (2013). Do scores on three commonly used measures of critical thinking correlate with academic success of health professions trainees? A systematic review and meta-analysis. *Academic Medicine*, *88*(5), 724-734. [10.1097/ACM.0b013e31828b0823](https://doi.org/10.1097/acm.0b013e31828b0823)

Rustamov, E., Nuriyeva, U. Z., Allahverdiyeva, M., Abbasov, T., & Rustamova, N. (2024). A structural equation modeling of academic locus of control, procrastination, and their impact on school satisfaction: Insights from the Azerbaijani educational system. International Journal of Educational Methodology, 10(1), 93-101. https://doi.org/10.12973/ijem.10.1.893

Shcheglova, I., Costley, J., Gorbunova, E., Lange, C. (2024). Does activation of higher-order thinking skills lead to students (dis) satisfaction with their academic experience?. *Innovations in Education and Teaching International*, 1-14. <https://doi.org/10.1080/14703297.2024.2332738>

Stupnisky, R.H., Renaud, R.D., Daniels, L.M. (2008) The Interrelation of First-Year College Students’ Critical Thinking Disposition, Perceived Academic Control, and Academic Achievement. *Res High Educ* 49, 513–530. <https://doi.org/10.1007/s11162-008-9093-8>

Tasgin, A., & Dilek, C. (2023). The mediating role of critical thinking dispositions between secondary school student's self-efficacy and problem-solving skills. *Thinking Skills and Creativity*, *50*, <https://doi.org/10.1016/j.tsc.2023.101400>

Uzun, K., & Karatas, Z. (2020). Predictors of Academic Self Efficacy: Intolerance of Uncertainty, Positive Beliefs about Worry and Academic Locus of Control. *International Education Studies*, *13*(6), 104-116.

Vu, T. V., Scharmer, A. L., van Triest, E., van Atteveldt, N., & Meeter, M. (2024). The reciprocity between various motivation constructs and academic achievement: a systematic review and multilevel meta-analysis of longitudinal studies. *Educational Psychology*, *44*(2), 136–170. https://doi.org/10.1080/01443410.2024.2307960

Yurt, E., & Hayli, Ç. M. (2025). Mediating role of self-efficacy and cognitive flexibility in the relationship between critical thinking and positive mental health in Turkish nursing students: a cross-sectional study. *BMJ open*, *15*(8), e097631. <https://doi.org/10.1136/bmjopen-2024-097631>

Yüce, E. (2023). Critical thinking, autonomous learning, and academic grit among preservice EFL teachers. *Thinking Skills and Creativity*, *50*, 101382. <https://doi.org/10.1016/j.tsc.2023.101382>
